# Trajectory of beta cell function and insulin clearance in stage 2 type 1 diabetes: natural history and response to teplizumab

**DOI:** 10.1007/s00125-024-06323-0

**Published:** 2024-11-19

**Authors:** Alfonso Galderisi, Emily K. Sims, Carmella Evans-Molina, Alessandra Petrelli, David Cuthbertson, Brandon M. Nathan, Heba M. Ismail, Kevan C. Herold, Antoinette Moran

**Affiliations:** 1 Department of Pediatrics, Yale University, New Haven, CT USA; 2 Department of Pediatrics, Center for Diabetes and Metabolic Diseases, Herman B Wells Center for Pediatric Research, Indiana University School of Medicine, Indianapolis, IN USA; 3 Department of Clinical Sciences and Community Health, University of Milan, Milan, Italy; 4 Pio Albergo Trivulzio, Milan, Italy; 5 Health Informatics Institute, University of South Florida, Tampa, FL USA; 6 Department of Pediatrics, University of Minnesota, Minneapolis, MN USA; 7 Department of Immunobiology, Yale University, New Haven, CT USA; 8 Department of Internal Medicine, Yale University, New Haven, CT USA

**Keywords:** Insulin clearance, Insulin secretion, Insulin sensitivity, Oral minimal model, Stage 2 type 1 diabetes, Teplizumab

## Abstract

**Aims/hypothesis:**

We aimed to analyse TrialNet Anti-CD3 Prevention (TN10) data using oral minimal model (OMM)-derived indices to characterise the natural history of stage 2 type 1 diabetes in placebo-treated individuals, to describe early metabolic responses to teplizumab and to explore the predictive capacity of OMM measures for disease-free survival rate.

**Methods:**

OMM-estimated insulin secretion, sensitivity and clearance and the disposition index were evaluated at baseline and at 3, 6 and 12 months post randomisation in placebo- and teplizumab-treated groups, and, within each group, in slow- and rapid-progressors (time to stage 3 disease >2 or $\le$ 2 years). OMM metrics were also compared with the standard AUC C-peptide. Percentage changes in CD8^+^ T memory cell and programmed death-1 (PD-1) expression were evaluated in each group.

**Results:**

Baseline metabolic characteristics were similar between 28 placebo- and 39 teplizumab-treated participants. Over 12 months, insulin secretion declined in placebo-treated and rose in teplizumab-treated participants. Within groups, placebo slow-progressors (*n*=14) maintained insulin secretion and sensitivity, while both declined in placebo rapid-progressors (*n*=14). Teplizumab slow-progressors (*n*=28) maintained elevated insulin secretion, while teplizumab rapid-progressors (*n*=11) experienced mild metabolic decline. Compared with rapid-progressor groups, insulin clearance significantly decreased between baseline and 3, 6 and 12 months in the slow-progressor groups in both treatment arms. In aggregate, both higher baseline insulin secretion (*p*=0.027) and reduced 12 month insulin clearance (*p*=0.045) predicted slower progression. A >25% loss of insulin secretion at 3 months had specificity of 0.95 (95% CI 0.86, 1.00) to identify rapid-progressors and correctly classified the 2 year risk for progression in 92% of participants, with a sensitivity of 0.19 (95% CI 0.08, 0.30). OMM-estimated insulin secretion outperformed AUC C-peptide to differentiate groups by treatment or to predict progression. Metabolic changes were paralleled by relative frequency of change in PD-1^+^ CD8^+^ T effector memory cells.

**Conclusions/interpretation:**

OMM measures characterise the metabolic heterogeneity in stage 2 diabetes, identifying differences between rapid- and slow-progressors, and heterogeneous impacts of immunotherapy, suggesting the need to account for these differences when designing and interpreting clinical trials.

**Graphical Abstract:**

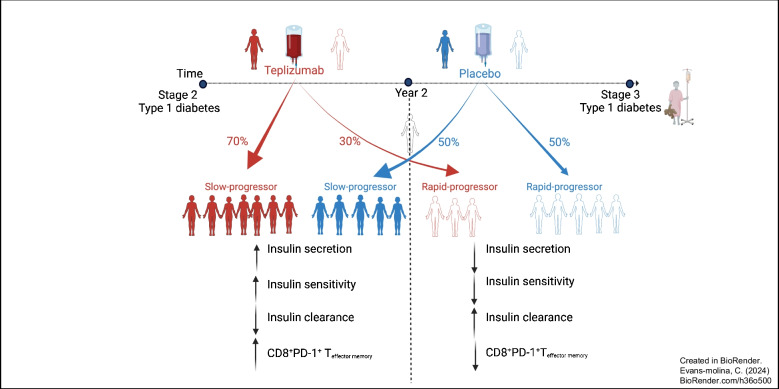

**Supplementary Information:**

The online version of this article (10.1007/s00125-024-06323-0) contains peer-reviewed but unedited supplementary material.


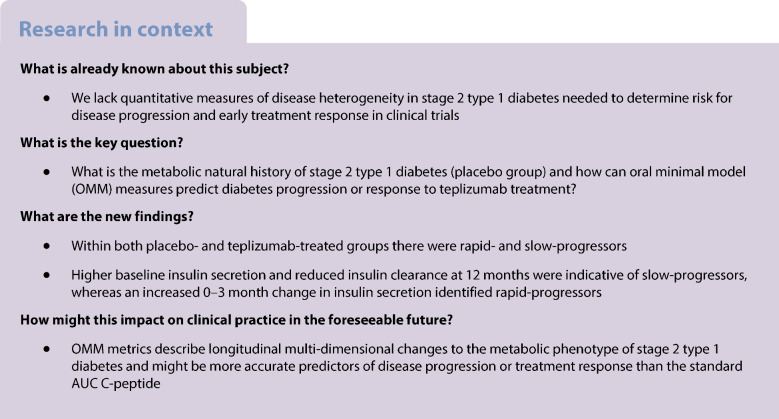


## Introduction

Type 1 diabetes begins with a presymptomatic phase defined as the presence of two or more islet autoantibodies without (stage 1) or with (stage 2) dysglycaemia [1]. Tracking progression to stage 3 (clinical) disease and quantifying the response to experimental therapies remains a challenge.

C-peptide AUC during an OGTT is the standard metric to describe changes in beta cell function in early type 1 diabetes [2–5], but it has limited ability to identify subtle metabolic changes which might be relevant to the risk of disease progression or to describe disease heterogeneity [6]. Additionally, it is unable to differentiate the insulin sensitivity (IS) and insulin clearance components of the metabolic phenotype, which may also impact progression [7]. Growing evidence suggests that metrics combining glucose, insulin and C-peptide, including Index60 or the Diabetes Prevention Trial Risk Score (DPTRS) [8], are more sensitive to metabolic changes in early type 1 diabetes [9–11], and that relative changes (vs absolute values) of these model-derived indices of beta cell function might have better discriminatory capability [11, 12].

The oral minimal model (OMM) is a validated methodology that uses C-peptide and insulin levels to estimate insulin secretion, sensitivity and clearance during an OGTT, allowing quantification of beta cell function and detection of subtle changes during disease progression [7, 12–14]. Its use of two-compartment kinetics to describe insulin secretion is more accurate than raw C-peptide data due to the nonlinear dynamic secretion trajectory during the OGTT [7, 15–17].

An accurate description of the metabolic phenotype in preclinical stages of type 1 diabetes may help inform approaches to disease-modifying treatments such as teplizumab. The TrialNet Anti-CD3 Prevention Trial (TN10) showed that a single course of teplizumab delayed progression to stage 3 (clinical) type 1 diabetes by 32.5 months in individuals with stage 2 disease [2, 18]. Teplizumab-treated individuals experienced early post-treatment increases in AUC C-peptide, while placebo-treated individuals showed progressive AUC C-peptide decline [18]. However, there was heterogeneity within both treatment groups, with ~30% of the people receiving teplizumab progressing to clinical diabetes within 2 years from treatment.

The objectives of this post hoc analysis of the TN10 dataset were to apply OMM-derived indices to characterise the natural history of the metabolic phenotype in stage 2 type 1 diabetes in placebo-treated individuals, and to describe the early metabolic response to teplizumab. We explored the clinical relevance and predictive capacity of OMM-estimated beta cell functional changes and insulin clearance within the first year of treatment with respect to long-term disease-free survival. T cell data provided a unique opportunity to characterise metabolo–immunophenotype relations.

## Methods

### Study design

In the TN10 placebo-treated group, median time to progression to stage 3 disease was 2 years. We defined slow- and rapid-progressors, regardless of treatment arm, as those who developed clinical disease after or before this median progression rate. Although not all participants progressed to stage 3 diabetes during the study, we considered the term ‘slow-progressors’ appropriate given the ~100% lifetime risk of progression to clinical disease amongst individuals with stage 2 diabetes [1]. Data from the first 12 months after placebo or teplizumab treatment were analysed because after that, particularly in the placebo group, participant numbers declined precipitously as individuals progressed to stage 3 disease.

### Participants

The protocol and results of TN10 have been reported (ClinicalTrials.gov registration no. NCT01030861) [2]. Formal approval was obtained from appropriate ethical review boards. Briefly, the trial enrolled relatives of people with type 1 diabetes who had stage 2 diabetes. After a baseline OGTT, participants were randomised to receive a single 14 day course of teplizumab or placebo. For the current post hoc analyses, only participants who completed both the baseline and 3 month OGTT were included. Data from 28 placebo- and 39 teplizumab-treated participants were analysed; excluded were four placebo- and three teplizumab-treated participants who did not complete the infusion and two teplizumab-treated participants who did not complete the 3 month OGTT. Participants' race and ethnicity were reported in the parent trial [2]. Due to the inclusion of 95% non-Hispanic people in this cohort, with 97% white participants, we were unable to evaluate whether the ethnic background could have affected this secondary analysis and whether our conclusion can be generalisable to other ethnic groups. Participants’ sex was self-reported.

### OGTT-based analyses

Participants underwent standard OGTTs with glucose, C-peptide and insulin levels measured every half hour for 2 h; OGTTs were performed at baseline and at 3 and 6 months post treatment, and every 6 months thereafter until diagnosis of stage 3 disease. The risk indices Index60 [6] and DPTRS [19] were calculated as described. OGTT-derived AUC C-peptide was computed using the trapezoidal rule.

OMM methodology was used to estimate total insulin secretion during the OGTT (Phi total [φ_total_]), IS, beta cell function (disposition index [DI], the product of φ_total_ and IS) and insulin clearance from OGTTs performed at baseline and at 3, 6 and 12 months [12]. The model was previously validated in children, adolescents and adults using multiple-tracer meal protocols and euglycaemic and hyperglycaemic clamps [14, 20, 21], and is described in detail by Cobelli et al [12].

φ_total_ is computed from 0, 30, 60, 90 and 120 min OGTT C-peptide and glucose concentrations, using changes from baseline and rates of change. Higher φ_total_ reflects greater insulin secretion over the entire OGTT. When the OMM includes 10 and 20 min samples, it can accurately dissect φ_total_ into dynamic and static components. Phi dynamic (φ_dynamic_) represents release of preformed insulin vesicles and depends on the rate of glucose increase [12]; it is roughly analogous to first-phase insulin secretion. Phi static (φ_static_) measures new insulin production in response to rising glucose concentrations and is similar to second-phase insulin secretion [22]. The absence of 10 and 20 min measures in this study protocol limited the accuracy of the relative contributions of the dynamic and static components to φ_total_ [22].

IS is derived from 0, 30, 60, 90 and 120 min insulin and glucose concentrations; lower IS indicates greater insulin resistance. DI represents the hyperbolic relationship between insulin secretion and sensitivity. Lower DI indicates reduced beta cell function and is driven by insulin secretion and/or sensitivity changes.

Insulin clearance is computed as the ratio of the 2 h OGTT AUC of the OMM-derived insulin secretion rate (ISR; AUC_ISR_) over AUC insulin; lower values represent lower clearance [23] with greater insulin retention in the circulation. Measures based on the ISR are more accurate than AUC C-peptide estimates [23]. While insulin and C-peptide are secreted in an equimolar ratio, more than 80% of secreted insulin, but not C-peptide, is cleared during the hepatic first pass, differentially reducing insulin concentrations in the systemic circulation [23]. AUC_ISR_ over AUC insulin represents whole body insulin clearance. Because the hepatic component is its major contributor [23], whole body changes are primarily driven by changes in hepatic insulin extraction.

### Flow cytometry

TN10 flow cytometry analyses have been described [2, 18]. Here, we evaluated differences in CD8^+^ T memory cell (CD3^+^CD8^+^CD56^−^CD45R0^+^) expression between placebo- and teplizumab-treated rapid- and slow-progressors in partially exhausted CD8^+^ T memory cells (Killer cell lectin-like receptor G1 [KLRG1]^+^ T-cell immunoreceptor with immunoglobulin and ITIM domain [TIGIT]^+^), which are associated with a positive clinical response to teplizumab in both stage 3 [24] and stage 2 type 1 diabetes [2, 18, 24, 25], and in expression of programmed death-1 (PD-1), a checkpoint molecule which helps maintain self-tolerance. PD-1 inhibition in cancer therapy is associated with development of islet autoimmunity [26, 27]. Expression of PD-1 was assessed in two subsets of CD8^+^ T memory cells, effector memory T (T_EM_) (C-C chemokine receptor type 7 negative [CCR7]^−^) and central memory T (T_CM_) cells (CCR7^+^). We also tested differential expression of these T cell phenotypes between those with high (>25% of baseline) and low φ_total_ loss at 3 months, independent of treatment arm.

### Statistical analysis

For each treatment arm, absolute values of insulin secretion, IS, DI, insulin clearance, DPTRS and Index60 as well as percentage change from baseline (*t*_0_) were quantified at 3, 6 and 12 months post treatment. A linear mixed model analysis compared trajectories for absolute values and percentage change over time. Proportional hazards regression and logrank tests were used for cumulative incidence analyses of stage 3 progression. The longitudinal metabolic phenotypes of slow- and rapid-progressor groups within each treatment arm were described.

To understand whether early changes in insulin secretion could predict subsequent course, diabetes progression rates were compared after stratifying the cohort by percentage change in insulin secretion from randomisation to the 3 month OGTT. Percentage change was used rather than absolute values to address baseline heterogeneity in insulin secretion and a relatively small sample size. An optimal cut-point analysis using Cox regression determined the threshold value that best predicted progression. Adjustments for multiple comparisons were not made due to limited sample size and the clinical rationale for the choice of comparison groups (rapid- vs slow-progressors). Results of paired comparisons are reported as unadjusted *p* values unless age and sex adjustments modified the significance of the observed differences.

Analyses were performed using STATA.13 software (StataCorp 2013; Stata Statistical Software: Release 13; College Station, TX, USA; StataCorp) and Prism 10.0 (GraphPad Software 2023, San Diego, CA, USA). The OMM was numerically identified by nonlinear least squares, as implemented in SAAM II v.2.3 (The Epsilon Group 2012–2013, Charlottesville, VA, USA).

## Results

### Baseline participant characteristics

#### Baseline metabolic characteristics were similar between placebo- and teplizumab-treated groups

At baseline, placebo- (*n*=28) and teplizumab-treated (*n*=39) groups had similar OMM indices, AUC C-peptide and risk indices of disease progression (Index60, DPTRS) (Table 1). Median (25th, 75th centile) time to stage 3 was 27.3 (7.9, 48) and 46.7 (22.3, 48) months for the placebo and teplizumab groups, respectively (*p*=0.014).

**Table 1:** Baseline characteristics

Characteristic	Placebo *N*=28			Teplizumab *N*=39			Placebo vs teplizumab
	Slow-progressor *N*=14	Rapid-progressor *N*=14	*p*	Slow-progressor *N*=28	Rapid-progressor *N*=11	*p*	*p*
Age (years)	14.8 (10.4, 17.9)	14.9 (12.2, 16.3)	0.430	14.9 (12.0, 35.1)	13.6 (11.4, 18.4)	0.146	0.252
BMI (kg/m^2^)	20.7 (17.7, 23.5)	21.5 (18.6, 25.4)	0.319	19.1 (17.5, 28.5)	19.3 (16.2, 22.8)	0.112	0.415
BMI *z* score	0.73 (0.39, 1.59)	0.84 (0.25, 1.08)	0.163	0.67 (−0.33, 1.66)	0.42 (−1.05, 1.38)	0.467	0.442
Female sex, *n* (%)	6 (43)	6 (43)	0.680	12 (43)	6 (55)	0.732	0.615
Fasting glucose (mmol/l)	5.2 (5.0, 5.8)	5.4 (4.8, 5.9)	0.452	5.2 (4.9, 5.4)	5.5 (5.2, 5.8)	0.063	0.466
1 h glucose (mmol/l)	9.7 (9.0, 11.3)	11 (9.3, 12.1)	0.054	10.2 (9.0, 11.8)	10.5 (9.8, 12.0)	0.194	0.337
2 h glucose (mmol/l)	7.9 (6.8, 9.2)	8.9 (8.0, 9.6)	0.038	8.6 (7.8, 9.5)	8.3 (7.8, 9.6)	0.427	0.345
Fasting C-peptide (nmol/l)	0.53 (0.40, 0.63)	0.59 (0.46, 0.79)	0.169	0.49 (0.36, 0.86)	0.53 (0.46, 0.59)	0.442	0.309
1 h C-peptide (nmol/l)	2.4 (1.6, 2.9)	1.9 (1.5, 2.4)	0.282	1.9 (1.6, 3.3)	1.8 (1.3, 2.0)	0.116	0.364
Index60	0.07 (−1.07, 0.79)	1.98 (1.48, 2.34)	0.010**	0.53 (−0.20, 1.21)	0.59 (0.24, 1.07)	0.189	0.417
DPTRS	9.2 (8.1, 9.4)	9.9 (8.7, 10.5)	0.009**	9.2 (8.4, 9.8)	9.7 (9.2, 10.0)	0.030*	0.933
AUC C-peptide (nmol/l×min)	249.5 (171.8, 322.3)	202.0 (185.9, 262.2)	0.170	211.9 (176.8, 311.1)	197.1 (168.0, 235.0)	0.134	0.441
Insulin secretion (φ_total_)	130.8 (94.2, 148.8)	82.9 (54.1, 108.1)	0.003**	113.6 (71.7, 146.7)	80.3 (62.6, 123.9)	0.099	0.475
Insulin secretion (φ_dynamic_)	1396.5 (874.0, 1642.3)	862.4 (676.4, 1396.6)	0.090	1001.8 (654.4, 1538.1)	890.6 (388.0, 1379.7)	0.280	0.283
Insulin secretion (φ_static_)	103.7 (73.1, 116.0)	80.3 (45.0, 125.3)	0.142	94.9 (56.0, 124.4)	68.3 (54.5, 78.6)	0.091	0.252
IS	0.98 (0.6, 2.2)	1.4 (0.6, 3.0)	0.297	1.2 (0.6, 2.0)	1.4 (1.1, 2.4)	0.230	0.305
DI	125.7 (97.1, 207.2)	113.3 (63.5, 243.0)	0.256	101.2 (63.0, 150.7)	106.1 (78.2, 204.4)	0.286	0.107
Insulin clearance (AUC ISR_C-peptide_/AUC_insulin_)	1.8 (1.2, 3.4)	1.2 (0.9, 2.5)	0.085	1.3 (0.9, 2.5)	2.0 (1.1, 2.6)	0.197	0.326

Data are expressed as median (25th, 75th centile), unless otherwise indicated

Kruskal–Wallis rank test and Dunn’s test were used for pairwise comparisons

*p<0.05, **p<0.01

#### Fifty per cent of placebo-treated and 28% of teplizumab-treated participants were identified as rapid-progressors (*p*=0.001)

The median time to stage 3 clinical disease was 5.8 (3.1, 12.0) and 18.6 (12.2, 22.3) months for placebo and teplizumab rapid-progressor groups, respectively (*p*<0.001). Within the four subgroups (Table 1), placebo rapid-progressors had worse baseline insulin secretion (φ_total_) than placebo slow-progressors (*p*=0.003) and teplizumab slow-progressors (*p*=0.017), but were similar to teplizumab rapid-progressors (*p*=0.123). Individual baseline components of φ_total_ (static and dynamic) did not differ between rapid- and slow-progressors in placebo and teplizumab groups (Table 1). The 2 h OGTT glucose (*p*=0.038), Index60 (*p*=0.010) and DPTRS (*p*=0.009) were all more favourable in placebo slow- vs placebo rapid-progressors, while IS, DI and insulin clearance did not differ.

Within the teplizumab group, slow- and rapid-progressors displayed similar baseline metabolic characteristics; the only significant difference was a lower DPTRS in slow-progressors (*p*=0.031), suggesting that overall, teplizumab treatment abrogated impacts of many of these baseline risk factors on rates of stage 3 progression.

### Twelve month longitudinal OMM-estimated metabolic trajectories

#### Longitudinal metabolic phenotypes over 12 months differed between placebo- and teplizumab-treated groups

**Fig. 1:**
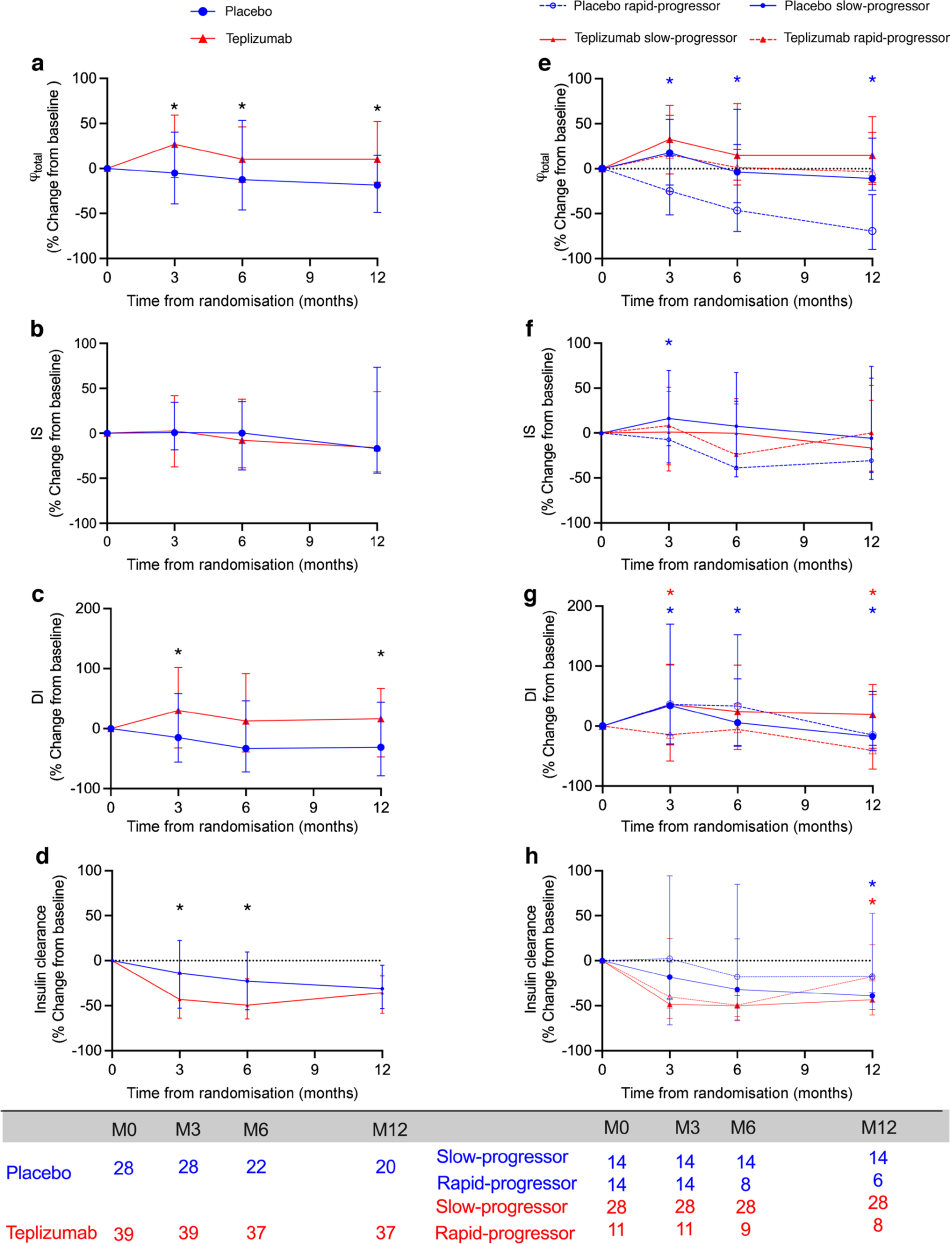
(**a**–**d**) Placebo vs teplizumab. Trajectories of the percentage change from baseline in (**a**) insulin secretion (φ_total_), (**b**) IS, (**c**) beta cell function (DI) and (**d**) insulin clearance. The asterisks indicate significant differences (*p*<0.05). (**e**–**h**) Placebo vs teplizumab by rapid- and slow-progressor status. Percentage change from baseline of (**e**) insulin secretion (φ_total_), (**f**) IS, (**g**) beta cell function (DI) and (**h**) insulin clearance. Rapid progression is defined as ≤2 years and slow progression >2 years to diagnosis of stage 3 diabetes. Data are expressed as median (25th, 75th centile) and the numbers of participants are shown in the table below. The red and blue asterisks indicate significant differences (*p*<0.05) for comparisons between rapid- and slow-progressors within the teplizumab and placebo groups, respectively, at each time point. M, month

The placebo group experienced a steady decline in insulin secretion and DI over 12 months. In contrast, insulin secretion rose in teplizumab-treated participants, peaking at 3 months, declining slightly by 6 months, then stabilising by 12 months (Fig. 1a–d, Table 2). The difference in insulin secretion trajectories between placebo and teplizumab groups was significant at 3 (*p*=0.021), 6 (*p*=0.043) and 12 months (*p*=0.005). Linear mixed model analysis of insulin secretion trajectories over 12 months confirmed differences in insulin secretion between the two groups (*p*=0.023). IS did not differ between treatment groups, remaining relatively constant overall. Driven by greater insulin secretion, the teplizumab group exhibited increased DI than the placebo group. Reduction in insulin clearance was more pronounced in the teplizumab than the placebo group over the first 6 months, but tended to converge at 12 months (*p*=0.002, linear mixed model).

**Table 2:** Longitudinal metabolic changes

Variable	Placebo *N*=28			Teplizumab *N*=39			Placebo (*n*=28) vs teplizumab (*n*=39)
	Slow-progressor	Rapid-progressor	*p*	Slow-progressor	Rapid-progressor	*p*	*p*
% Change from baseline M3	*N*=*14*	*N*=*14*		*N*=*28*	*N*=*11*		
Insulin secretion (φ_total_)	+17.7 (−15.4, +54.8)	−26.4 (−57.5, +11.7)	0.013*	+37.1 (−5.9, +59.2)	+10.0 (−23.3, +68.1)	0.231	0.021*
Insulin secretion (φ_dynamic_)	−12.4 (−41.4, +45.6)	−45.7 (−67.9, −2.6)	0.117	+44.5 (−0.1, +168.4)	+13.7 (−23.9, +15.5)	0.086	<0.001***
Insulin secretion (φ_static_)	+21.2 (−16.8, +84.2)	−21.2 (−63.0, +14.4)	0.018*	+15.8 (−4.7, +49.9)	+0.85 (−25.0, +85.8)	0.213	0.003
IS	+30.8 (−14.0, +69.7)	−8.8 (−30.7, +13.2)	0.047*	−5.0 (−35.2, +46.2)	+13.9 (−40.7, +40.3)	0.363	0.452
Beta cell function (DI)	+43.3 (−14.8, +169.4)	−43.2 (−68.5, −14.7)	0.002**	+36.0 (−31.2, +102.7)	+11.4 (−54.6, +102.0)	0.267	0.043*
AUC C-peptide	+1.1 (−13.0, +13.5)	−12.9 (−18.3, −2.0)	0.065	+6.3 (−9.1, +26.9)	+4.8 (−14.1, +34.3)	0.729	0.033*
Insulin clearance (AUC ISR_C-peptide_/AUC_insulin_)	−18.3 (−69.8, −3.2)	−0.12 (−47.2, +50.9)	0.105	−47.1 (−64.2, −17.0)	−39.5 (−47.0, −17.8)	0.155	0.055
DPTRS	−4.4 (−12.6, −0.3)	+9.9 (−0.9, +22.9)	0.002**	−2.6 (−8.4, +3.2)	−4.1 (−14.2, +9.3)	0.499	0.106
Index60	+7.7 (−111.3, +142.2)	+42.4 (+16.1, +125.7)	0.087	−42.6 (−72.4, +3.4)	−52.4 (−77.2, +25.7)	0.400	<0.001
% Change from baseline M6	*N*=*14*	*N*=*8*		*N*=*28*	*N*=*9*		
Insulin secretion (φ_total_)	+15.8 (−39.4, +66.0)	−33.7 (−57.8, −3.8)	0.083	+17.1 (−10.2, +69.4)	+1.5 (−18.9, +23.7)	0.097	0.043*
Insulin secretion (φ_dynamic_)	+0.8 (−47.8 +67.1)	−38.6 (−64.8, +30.2)	0.098	−0.5 (−23.5, +66.8)	−8.6 (−23.6, +27.5)	0.245	0.315
Insulin secretion (φ_static_)	+12.3 (−35.0, +56.0)	−38.3 (−63.0, +8.9)	0.085	+17.0 (−12.6, +74.5)	+0.2 (−13.0, +27.8)	0.213	0.003
IS	+6.1 (−25.0, +25.8)	−16.5 (−41.2, +38.5)	0.328	−4.7 (−37.9, +38.4)	−11.6 (−34.8, +21.3)	0.466	0.405
Beta cell function (DI)	+5.7 (−43.4, +78.7)	−72.2 (−74.2, −26.0)	0.019*	+29.1 (−39.0, +111.5)	−15.7 (−38.6, +30.5)	0.190	0.093
AUC C-peptide	−5.8 (−18.3, +11.2)	−12.9 (−18.4, −2.0)	0.752	+6.3 (−9.1, +26.9)	+4.8 (−14.1, +34.3)	0.591	0.002**
Insulin clearance (AUC_C-peptide_/AUC_insulin_)	−31.9 (−54.5, +9.7)	−18.0 (−30.4, +2.1)	0.239	−50.0 (−64.7, −39.4)	−49.4 (−59.9, +21.9)	0.304	0.032*
DPTRS	−3.1 (−14.6, +4.3)	+26.7 (−7.2, +35.2)	0.017*	−1.5 (−11.6, +6.8)	+1.1 (−3.9, +7.8)	0.192	0.729
Index60	+10.8 (−72.3, +65.3)	+103.4 (+19.1, +155.4)	0.031	−33.0 (−87.5, +24.1)	−3.5 (−14.7, +10.6)	0.147	0.015
% Change from baseline M12	*N*=*14*	*N*=*6*		*N*=*28*	*N*=*8*		
Insulin secretion (φ_total_)	−10.9 (−28.9, +34.0)	−61.4 (−88.1, −24.3)	0.095	+20.3 (−11.4, +61.8)	−5.8 (−31.1, +10.2)	0.039*	0.005**
Insulin secretion (φ_dynamic_)	−26.4 (−60.4, +21.4)	−22.7 (−63.3, −14.0)	0.438	+8.6 (−31.8, +67.7)	−28.4 (−47.5, +5.7)	0.114	0.050
Insulin secretion (φ_static_)	−10.3 (−22.7, +33.6)	−71.8 (−92.7, −20.0)	0.012	+15.6 (−3.5, +58.2)	+3.2 (−26.1, +7.4)	0.067	0.007
IS	−11.0 (−44.4, +24.4)	−23.6 (−44.8, +122.5)	0.369	−16.5 (−43.9, +41.1)	+2.9 (−29.5, +53.0)	0.275	0.462
Beta cell function (DI)	−20.1 (−50.4, +50.7)	−79.1 (−88.8, +26.8)	0.034*	+17.7 (−40.5, +55.4)	−22.0 (−58.9, +59.1)	0.153	0.048*
AUC C-peptide	−28.8 (−52.5, +5.9)	+20.0 (−18.9, +68.5)	0.115	+6.4 (−31.2, +56.2)	−0.7 (−31.6, +74.6)	0.945	0.372
Insulin clearance (AUC_C-peptide_/AUC_insulin_)	−37.7(−53.7, −12.7)	−14.3 (−20.9, +50.6)	0.043*	−48.1 (−60.7, −27.6)	−10.6 (−46.2, +17.7)	0.029*	0.062
DPTRS	−2.9 (−7.5, +5.4)	+32.2 (+11.1, +44.0)	0.001**	−3.9 (−9.3, +7.5)	+2.9 (−4.6, +9.8)	0.138	0.086
Index60	12.2 (−40.3, +129.8)	+139.0 (−83.5, +461.2)	0.282	−8.5 (−60.0, +81.1)	+8.1 (−7.2, +28.7)	0.252	0.209

Data are expressed as median (25th, 75th centile)

Kruskal–Wallis rank test and Dunn’s test were used for pairwise comparisons

*p<0.05, **p<0.01, ***p<0.001

M, month

#### Within the placebo group, slow- and rapid-progressors exhibited different longitudinal metabolic trajectories in insulin secretion, DI and IS

Placebo slow-progressors demonstrated relatively preserved insulin secretion with modest fluctuation over 6 months and a slight decline to just below baseline by 12 months. Placebo rapid-progressors had a steady decline in insulin secretion (Table 2, Fig. 1e–h). DI followed similar patterns. IS increased during the first 3 months in placebo slow-progressors (*p*=0.047), followed by a gradual fall to slightly below baseline by 12 months. A steady reduction in IS was seen in placebo rapid-progressors at months 3, 6 and 12.

**Fig. 2:**
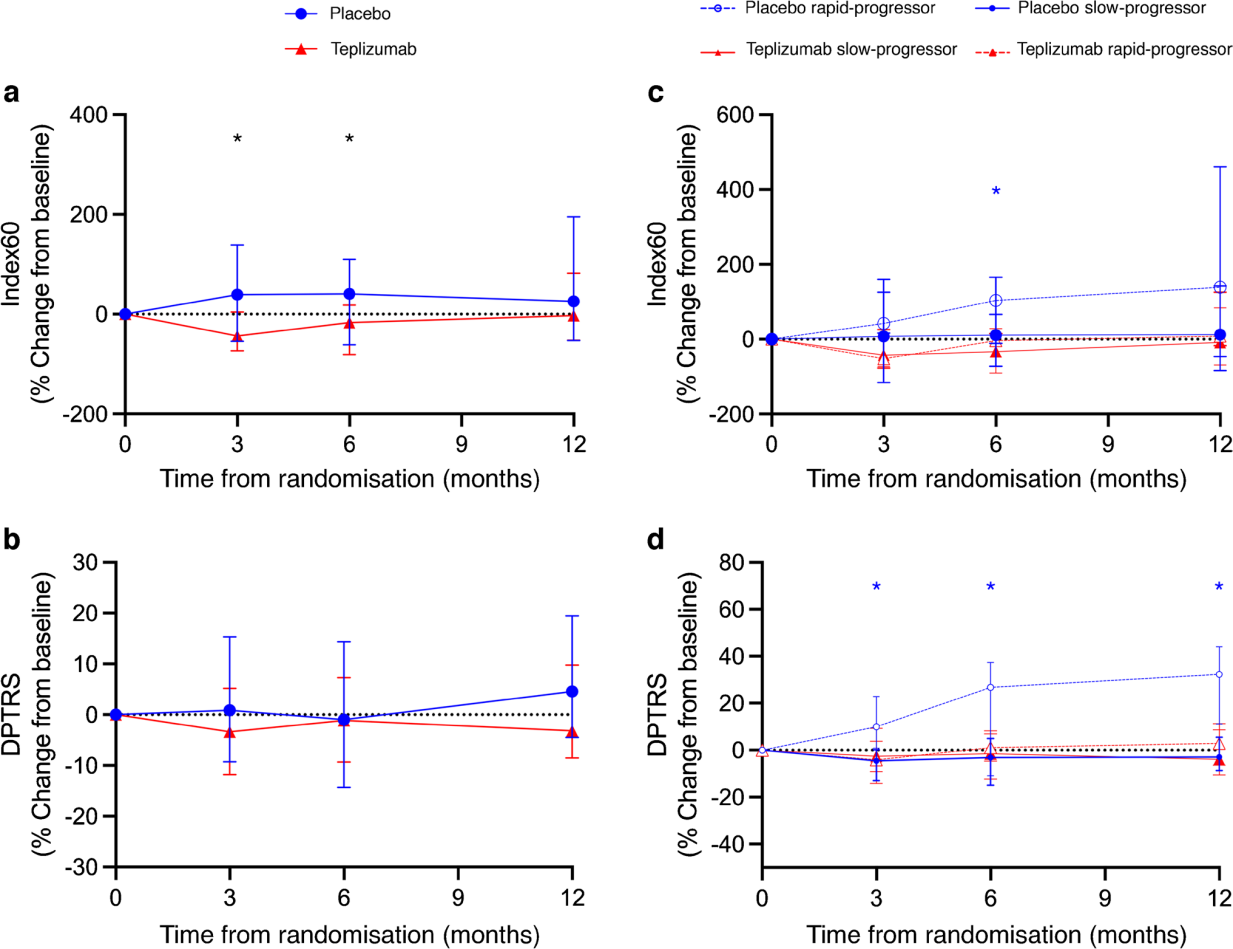
(**a**, **b**) Placebo vs teplizumab. Trajectories of the percentage change from baseline in (**a**) Index60 and (**b**) DPTRS. The asterisks indicate significant differences (*p*<0.05). (**c**, **d**) Placebo vs teplizumab by rapid- and slow-progressor status. Percentage change from baseline of (**c**) Index60 and (**d**) DPTRS. Rapid progression is defined as ≤2 years and slow progression >2 years to diagnosis of stage 3 diabetes. The number of participants evaluated at each time point is reported in Fig. 1. Data are expressed as median (25th, 75th centile)*.* The blue asterisks indicate significant differences (*p*<0.05) for comparisons between rapid- and slow-progressors within the placebo group at each time point

While relative changes in Index60 did not differ between rapid- and slow-progressors, the median DPTRS increased up to 30% from baseline in rapid-progressors at 12 months while remaining almost identical to baseline (−3%) in slow-progressors (Table 2, Fig. 2c, d).

#### Within the teplizumab-treated group, slow- and rapid-progressors exhibited different longitudinal metabolic trajectories in insulin secretion and DI, but not IS

In teplizumab slow-progressors, secretion peaked at 3 months (+37% from baseline, *p*=0.231 vs rapid-progressors), then fell modestly and stabilised (+17% from baseline at 6 months, *p*=0.097; +20% at 12 months, *p*=0.039). This was accompanied by corresponding increases in DI. Teplizumab rapid-progressors demonstrated a slight increase (+10%) in insulin secretion at 3 months, followed by a drop to just below baseline by 12 months. This pattern of relative stability was mirrored by DI. No significant changes in IS were noted in either teplizumab subgroup (Fig. 1e–h, Table 2). Linear mixed model analysis of the 12 month trajectories of OMM-estimated insulin secretion changes confirmed differential trends within each treatment group for slow- and rapid-progressors who received either placebo (*p*=0.003) or teplizumab (*p*=0.019).

Longitudinal changes in Index60 and DPTRS did not differ between rapid- and slow-progressors over 12 months, with an increasing trend in both groups (Table 2, Fig. 2c, d).

#### Slow-progressors demonstrated significantly enhanced ability to reduce insulin clearance compared with rapid-progressors, independent of treatment arm

Insulin clearance significantly decreased between baseline and 3, 6 and 12 months in both slow-progressor groups (by 12 months: placebo −37.7% from baseline, *p*=0.042; teplizumab −48.1%, *p*<0.001) (Table 2, Fig. 1h). In contrast, there was a more modest and not statistically significant change in insulin clearance from baseline over the year in both rapid-progressor groups (placebo −14.3%, *p*=0.844; teplizumab −10.6%, *p*=0.359). Interestingly, between baseline and 3 months, insulin clearance dropped rapidly in both teplizumab-treated groups, but while it stabilised by 12 months at this lower level in slow-progressors, this protective adaptation was only transient in teplizumab rapid-progressors.

### Predictive value of metabolic parameters

Higher OMM-derived insulin secretion was a baseline predictor of slower disease progression. For the overall study group, higher baseline φ_total_ decreased the hazard for progression (HR 0.984 [95% CI 0.974, 0.994], *p*=0.002), with a 26% increase in disease risk per each 10 unitary decrease in insulin secretion. Baseline IS and DI did not change the hazard for disease progression (*p*=0.153 and *p*=0.295).

Because peak insulin secretion occurred at 3 months, the predictive value of OMM metabolic variable changes from baseline to 3 months was tested. For the entire cohort, a 0–3 month increase in insulin secretion was associated with reduced risk for disease progression (*p*=0.027). We further explored the sensitivity and the specificity of different threshold values for the 3 month change in insulin secretion and identified 25% loss as the value with the highest specificity using a receiver operating characteristic (ROC) curve analysis. A φ_total_ loss >25% from baseline (‘high-loss’) had specificity of 0.95 (95% CI 0.86, 1.00) to identify those who progressed to stage 3 by 2 years.

**Fig. 3:**
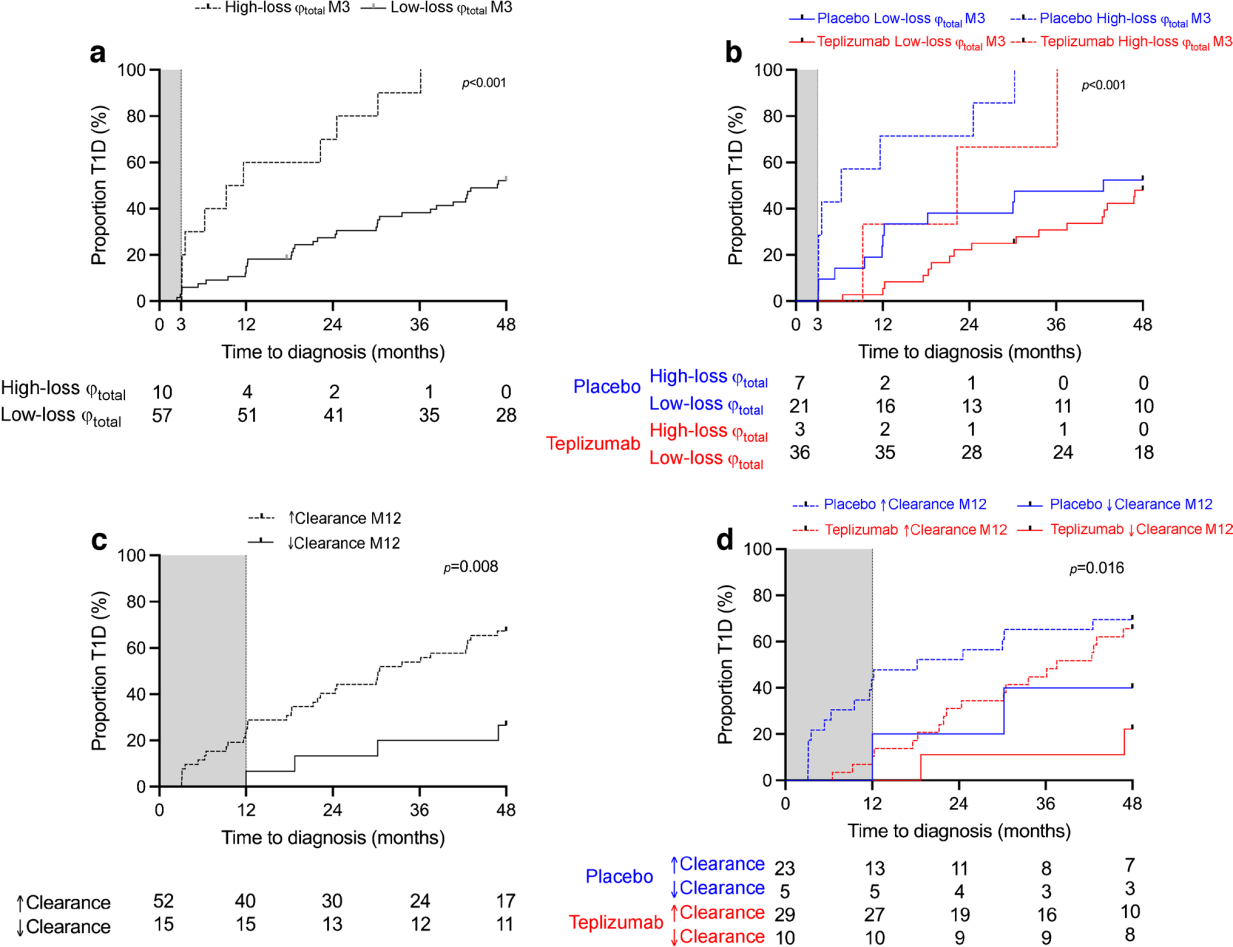
Kaplan–Meier estimates of the proportion of participants who progressed to stage 3 type 1 diabetes based on (**a**) >25% loss (high-loss) or ≤25% loss (low-loss) in insulin secretion (φ_total_) between baseline and 3 months across all participants (placebo + teplizumab), and (**b**) divided by treatment arm and progressor status or (**c**) based on an increase or a decrease in insulin clearance between baseline and 12 months across all participants (placebo + teplizumab), or (**d**) divided by treatment arm and progressor status. M, month; T1D, type 1 diabetes

As displayed in Fig. 3a, in the ten participants with high-loss insulin secretion at 3 months, 40% developed stage 3 disease by 12 months and 80% by 24 months. In contrast, in the 57 participants with low-loss insulin secretion at 3 months (≤25% reduction), only 10% progressed to stage 3 by 12 months and 28% by 24 months. Similar trends were observed within both treatment arms (Fig. 3b).

Despite the highly specific predictive value of loss of >25% in φ_total_ from baseline, the sensitivity of this measure was only 0.19 (95%CI 0.08, 0.30), regardless of treatment arm. The 3 month changes in φ_static_, the component of φ_total_ corresponding to second-phase insulin secretion, were not associated with reduced progression risk (*p*=0.100). Because of the absence of early insulin samples, the accuracy of φ_dynamic_ estimates was limited and we did not include this metric in the current analysis. Percentage changes in IS and DI at 3 months were not predictors of disease progression on a Cox regression model (*p*=0.796 and *p*=0.115).

While changes in insulin clearance did not differentiate the treatment groups at 3 months, reduction in insulin clearance at 12 months was associated with reduced risk of clinical disease progression (Fig. 3c, d). Those whose 12 month insulin clearance value was lower than baseline had half the risk for 2 year progression than those whose insulin clearance increased (HR 0.57 [95% CI 0.332, 0.981], *p*=0.045). Of 15 participants whose 12 month insulin clearance decreased from baseline, only two (13%) progressed to stage 3 disease by 24 months, 20% by 36 months and 27% by 48 months. In contrast, of 52 participants whose 12 month insulin clearance increased from baseline, 42% progressed by 24 months, 54% by 36 months and 67% by 48 months. As displayed in Fig. 3d, reduction of insulin clearance was associated with prolonged progression-free survival within both teplizumab and placebo groups.

### OMM-estimated insulin secretion outperformed AUC C-peptide to differentiate groups by treatment or to predict progression

While absolute values and percentage change of OMM-estimated insulin secretion differentiated placebo- from teplizumab-treated individuals at each time point, AUC C-peptide was less consistent, differentiating treatment groups at 6 (*p*=0.043) but not 3 (*p*=0.115) or 12 (*p*=0.291) months. AUC C-peptide percentage change from baseline differentiated treatment groups at 3 (*p*=0.033) and 6 (*p*=0.002) but not 12 months (*p*=0.372) (Table 2). Notably, no baseline differences in AUC C-peptide were present, nor did AUC C-peptide percentage change differ at any specified time points between rapid- and slow-progressors within either treatment group (Table 2), and change in AUC C-peptide at 3 months was not associated with differences in disease progression (*p*=0.347).

### Immunophenotype trajectories in rapid- and slow-progressors and relation to φ_total_

**Fig. 4:**
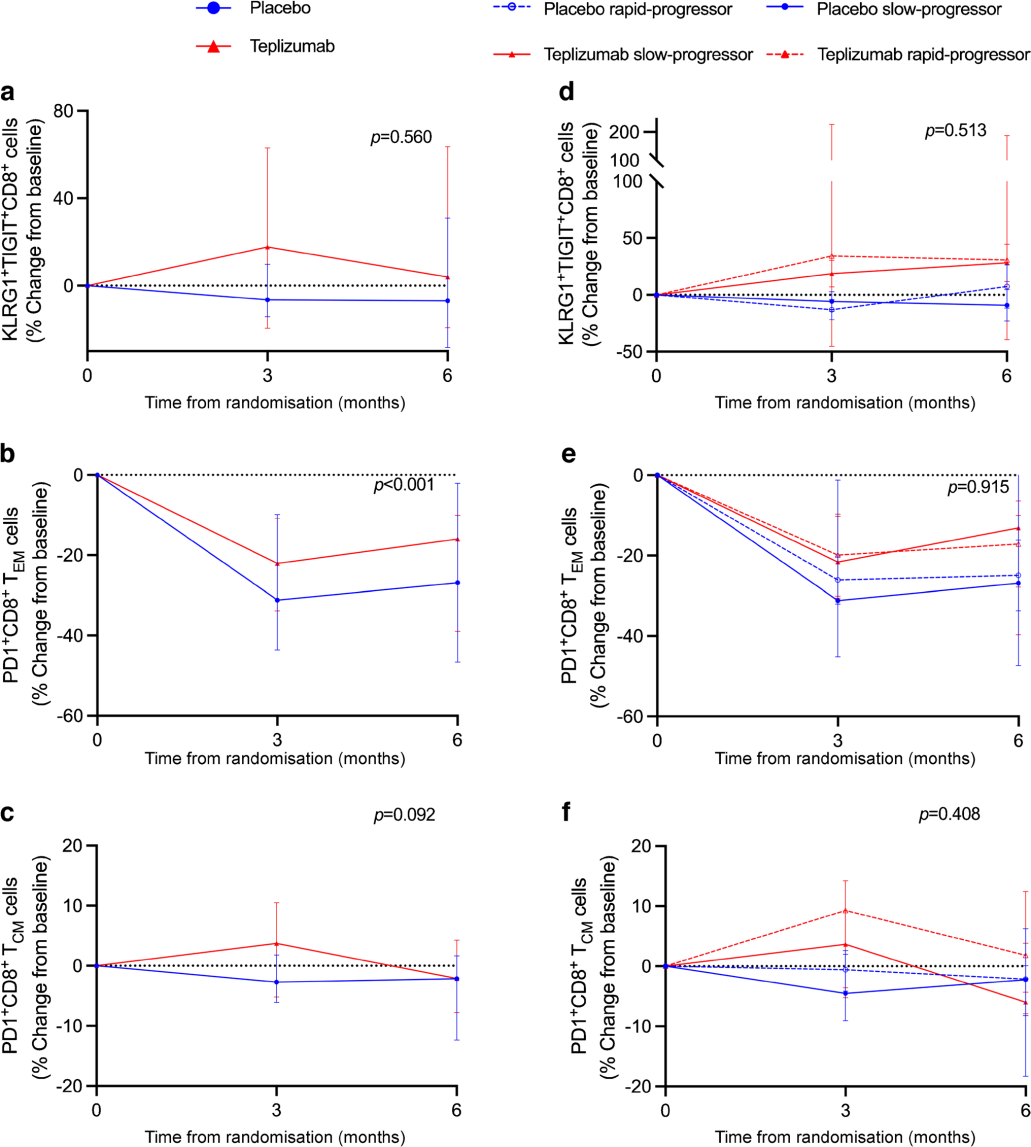
(**a**–**c**) Placebo vs teplizumab. Trajectories of the percentage change from baseline in (**a**) partially exhausted CD8^+^ T cells (KLRG1^+^TIGIT^+^), (**b**) CD8^+^ T_EM_ cells (CD3^+^CD56^−^CD8^+^CD45R0^+^CCR7^−^PD-1^+^) and (**c**) T_CM_ cells (CD3^+^CD56^−^CD8^+^CD45R0^+^CCR7^+^PD-1^+^). Trajectories have been compared using linear mixed-model analysis. (**d**–**f**) Placebo vs teplizumab by rapid- and slow-progressor status. Percentage change from baseline of (**d**) partially exhausted CD8^+^ T cells (KLRG1^+^TIGIT^+^), (**e**) CD8^+^ T_EM_ cells (CD3^+^CD56^−^CD8^+^CD45R0^+^CCR7^−^PD-1^+^) and (**f**) T_CM_ cells (CD3^+^CD56^−^CD8^+^CD45R0^+^CCR7^+^PD-1^+^). Rapid progression is defined as ≤2 years and slow progression >2 years to diagnosis of stage 3 diabetes. Trajectories have been compared using linear mixed-model analysis. Data are expressed as median (25th, 75th centile). The number of participants evaluated at each time point is reported in Fig. 1

At baseline, distribution of partially exhausted CD8^+^ T memory cells (CD3^+^CD8^+^CD56^−^ CD45R0^+^KLRG1^+^TIGIT^+^) did not differ between placebo or teplizumab groups (*p*=0.134) or between rapid- and slow-progressors within each group (placebo, *p*=0.166; teplizumab, *p*=0.489). As expected, this T cell subset transiently increased from baseline to 3 months in those treated with teplizumab but not placebo (*p*=0.030); by 6 months the change from baseline did not differ between the two treatment groups or the four subgroups (*p*=0.560 and 0.513, linear mixed model) (Fig. 4a, d).

Baseline distribution of CD8^+^ T_EM_ (CD3^+^CD56^−^CD8^+^CD45R0^+^CCR7^−^) PD-1^+^ and CD8^+^ T_CM_ (CD3^+^CD56^−^CD8^+^CD45R0^+^CCR7^+^) PD-1^+^ cells did not differ between teplizumab and placebo groups (*p*=0.177) or the four subgroups (*p*=0.994) (Fig. 4b, e). PD-1 expression in CD8^+^ T_EM_ cells decreased from baseline to 3 months in both groups, but the decline was less (more favourable) in the teplizumab-treated group (*p*<0.001). A similar pattern was seen in the subgroups. Significant differences were not observed in T_CM_ CD8^+^PD-1^+^ expression between the groups or subgroups (*p*=0.092 and *p*=0.408, linear mixed model) (Fig. 4b, e).

Those with high-loss insulin secretion between baseline and 3 months (>25% of baseline, *N*=10) displayed greater reduction of PD-1^+^CD8^+^ T_EM_ than the 57 participants with low-loss (median [IQR]) (−42.3% [−49.8%, −27.3%] vs −22.6% [−35.0%, −8.3%], *p*=0.023). No significant differences were observed for PD-1^+^CD8^+^ T_CM_ (*p*=0.790) or TIGIT^+^KLRG1^+^CD8^+^ T cells (*p*=0.397) (see electronic supplementary material [ESM] Fig. 1).

## Discussion

Using data from the TrialNet TN10 study, we report the first use of the OMM to characterise the natural history of the metabolic phenotype of stage 2 type 1 diabetes, as well as the effect of a single course of teplizumab. OMM indices demonstrated metabolic heterogeneity in both placebo- and teplizumab-treated individuals, characterised by differential dynamics of insulin secretion, sensitivity and clearance. Stable or increased insulin secretion over the first few months of observation and a reduction in insulin clearance were features of delayed disease progression.

In the current analyses, participants within the placebo and teplizumab treatment groups were characterised as slow- or rapid-progressors according to whether stage 3 developed after or before the placebo median of 2 years. Over the first study year, placebo-treated slow-progressors maintained relatively stable insulin secretion, DI and IS. In contrast, placebo rapid-progressors had steady declines in all three parameters. The placebo group exemplifies the natural history of stage 2 type 1 diabetes. Slow- and rapid-progressors may represent different phenotypes of early diabetes, or may simply reflect earlier vs more advanced phases of the disease process.

We report for the first time the longitudinal relationship between insulin clearance and insulin secretion in stage 2 diabetes. In both placebo- and teplizumab-treated individuals, insulin clearance decreased more in slow- vs rapid-progressors. In a setting of reduced insulin secretion, reduced insulin clearance is beneficial, allowing persistence of higher circulating insulin levels. Inadequacy of this compensatory mechanism in rapid-progressors might have reflected an underlying physiological difference allowing faster disease progression, or it may simply have been a manifestation of more advanced disease. Our group previously reported reduced insulin clearance in youth with stage 1 type 1 diabetes compared with non-diabetic control individuals in a cross-sectional analysis [11]. Decreased insulin clearance in the setting of lower endogenous insulin secretion has also been described in youth and adults with obesity and prediabetes [28], and in type 2 diabetes [29]. It was found in a porcine model of alloxan-induced reduction in beta cell mass [30], suggesting a metabolic adaptation to diminished beta cell numbers or insulin levels.

The role of insulin clearance in type 1 diabetes progression remains largely unexplored. The key regulator of hepatic insulin clearance is the transmembrane protein carcinoembryonic antigen-related cell adhesion molecule 1 (CEACAM1) [31], which promotes insulin endocytosis and degradation. CEACAM1 is also expressed by CD4^+^ T cells where it is upregulated by the proinflammatory cytokines IL-7 and IL-15 and by the mixed pro- and anti-inflammatory cytokine IL-2, as well as by the activated T cell receptor (TCR)–CD3 complex [32]. We hypothesise that autoimmune inflammation associated with type 1 diabetes leads to increased expression of the hepatic CEACAM1, increasing insulin clearance in rapid disease progressors. In contrast, teplizumab may interfere with CEACAM1 expression in both hepatocytes and T cells, resulting in both reduced insulin clearance and mitigation of the autoimmune process [32]. The dual role of CEACAM1 in autoimmune progression and hepatic insulin clearance deserves further investigation.

OMM-estimated insulin secretion, measured both as absolute φ_total_ values and as percentage change from baseline, outperformed AUC C-peptide in differentiating placebo and teplizumab treatment groups during the first TN10 study year. Furthermore, >25% loss of φ_total_ between baseline and 3 months was highly predictive of rapid progression regardless of treatment group. But despite 95% specificity, the sensitivity of this predictive measure was only 19%. Declining first-phase insulin secretion is a key predictor of type 1 diabetes progression [33]. The absence of 10 and 20 min time points in this study reduces the accuracy of estimates of the relative contributions of φ_dynamic_ and φ_static_ to φ_total_.

Our findings support non-redundant complimentary roles for OMM-derived indices and classical risk metrics such as Index60 and DPTRS. While both OMM-derived metrics and DPTRS differentiated rapid- and slow-progressors at baseline and over the 12 month period in the natural history of the disease (placebo group), insulin secretion and clearance outperformed DPTRS or Index60 at distinguishing rapid- and slow-progressors in the teplizumab group 12 months after treatment. Observations in larger longitudinal cohorts are necessary to compare the OMM metrics and the classical risk indices when used as predictors of disease progression or as early endpoints to quantify treatment response.

Teplizumab-induced partial exhaustion of CD8^+^ T memory cells (KLRG1^+^TIGIT^+^) has been associated with a positive clinical response to teplizumab in stage 2 type 1 diabetes [24, 25]. We expand on these findings by demonstrating that the percentage of cells with this exhausted phenotype increased in both slow- and rapid-progressor teplizumab-treated subgroups. As expected, this response was drug specific; placebo-treated participants had no change in the percentage of KLRG1^+^TIGIT^+^ cells.

T_EM_ cells express homing receptors for inflammatory tissue and play an important role in the autoimmune response. Reduction in the percentage of CD8^+^ T_EM_ cells expressing the inhibitory immune checkpoint PD-1 protein is associated with progression to clinical type 1 diabetes [34]. While CD8^+^ T_EM_ PD-1 expression decreased in all four subgroups, the decline was less in teplizumab-treated individuals. Although the numbers were small, those with an insulin secretion drop >25% from baseline to 3 months (7/28 participants in the placebo group, 3/39 teplizumab) exhibited a lower (less favourable) percentage of CD8^+^ T_EM_ PD-1^+^ cells compared with participants with ≤25% loss.

Major strengths of this study include the relatively large and richly characterised population of individuals with stage 2 type 1 diabetes followed longitudinally without teplizumab treatment. New use of OMM-derived indices allowed longitudinal assessment of insulin secretion, IS, DI and insulin clearance, and comparison with standard metrics such as AUC C-peptide. The role of insulin clearance as a major component of the metabolic phenotype of rapid- and slow-progressors is a novel finding and may lead to further investigations to identify the underlying mechanisms. The inclusion of the CD8^+^ T_EM_ PD-1^+^ subset provides preliminary evidence for parallel changes in metabolic and immune phenotypes.

Study limitations include the relatively small numbers within subgroups, and that analyses were not carried out beyond 12 months because of diminishing subgroup participants. Reduced numbers at 6 and 12 months may have underpowered subgroup analyses at these time points. OGTTs were not performed after stage 3 diagnosis, resulting in a more precipitous dropout from analysis in rapid-progressors. The lack of 10 and 20 min OGTT samples precluded accurate OMM-based estimation of early insulin secretion. However, this limitation was present in both of the randomised groups and thus does not diminish findings of between-group differences. We lacked the numbers to explore the relation between IS and age or pubertal status.

In conclusion, OMM indices elucidated heterogeneity of insulin secretion in stage 2 type 1 diabetes, identifying differences between rapid- and slow-progressor metabolic phenotypes within both the placebo- and teplizumab-treated groups, and suggesting the need to account for these differences when designing clinical trials. TrialNet has recently added 10 and 20 min samples to OGTT protocols to allow OMM assessment of early insulin secretion. Further study is required to assess the generalisability of our findings, validate proposed prediction thresholds and explore novel approaches to the design of clinical trials with combined use of OMM and immune markers.

## Supplementary Information

Below is the link to the electronic supplementary material.

ESM Fig (PDF 95 KB)

## Data Availability

The datasets generated during and/or analysed in the current study are available from the corresponding author upon reasonable request.

## Abbreviations

φ_dynamic_: Phi dynamic (early insulin secretion)
φ_static_: Phi static (late insulin secretion)
φ_total_: Phi total (total insulin secretion)
CEACAM1: Carcinoembryonic antigen-related cell adhesion molecule 1
CCR7: C-C chemokine receptor type 7
DI: Disposition index
DPTRS: Diabetes Prevention Trial Risk Score
IS: Insulin sensitivity
ISR: Insulin secretion rate
KLRG1: Killer cell lectin-like receptor G1
OMM: Oral minimal model
PD-1: Programmed death-1
T_CM_ cell: Central memory T cell
T_EM_ cell: Effector memory T cell
TIGIT: T-cell immunoreceptor with immunoglobulin and ITIM domain
TN10: TrialNet Anti-CD3 Prevention Trial in Stage 2 disease
