# Supplementary material for: Trajectory of beta cell function and insulin clearance in stage 2 type 1 diabetes: natural history and response to teplizumab
Source: Diabetologia. 2024 Nov 19;68(3):646–61. doi: 10.1007/s00125-024-06323-0 (PMC11832608; doi:10.1007/s00125-024-06323-0)
Supplement: Supplementary file 1 — ESM Fig (PDF 95 KB) [file 125_2024_6323_MOESM1_ESM.pdf]

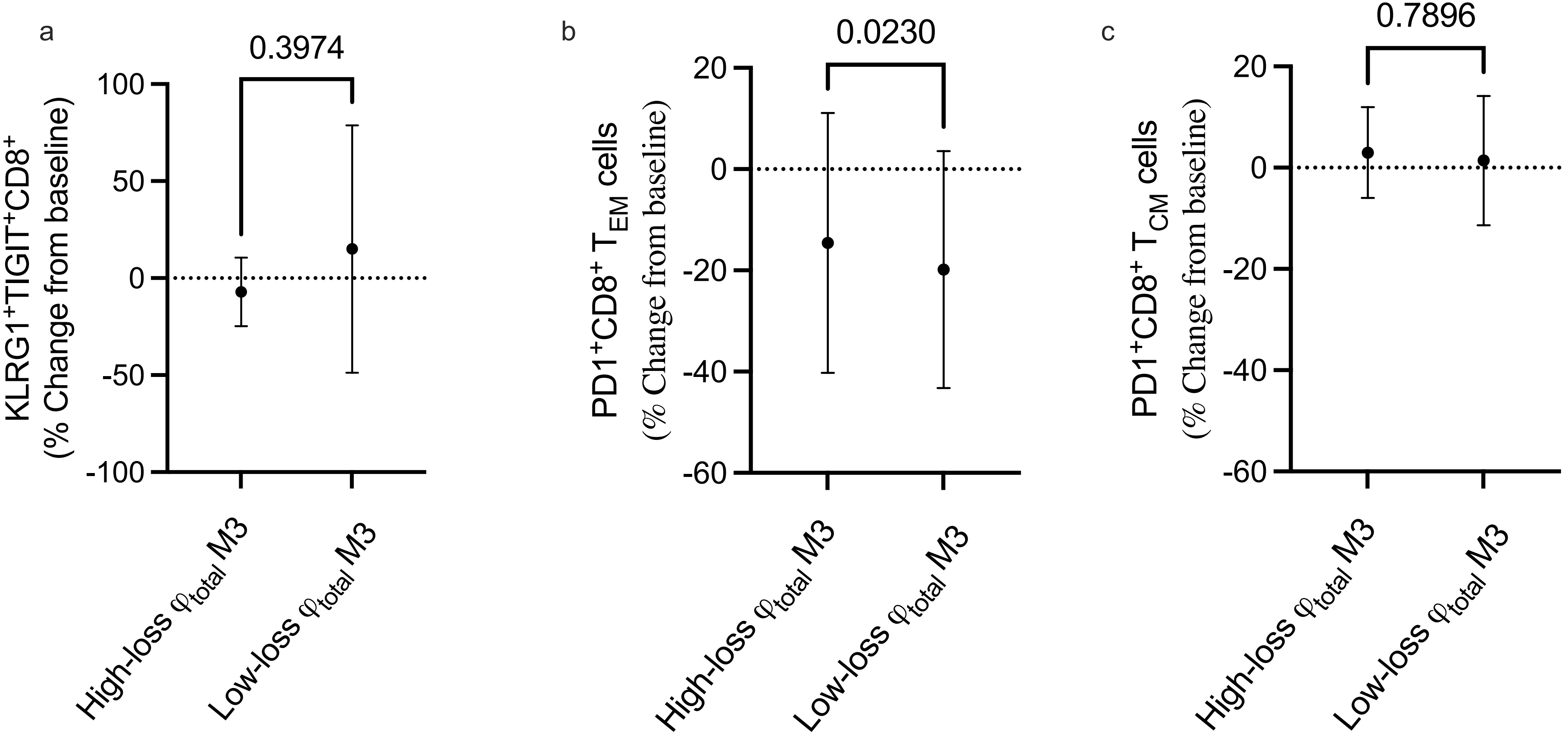

ESM Fig. 1 High-loss vs low-loss insulin secretion (φ_{total}) in the entire cohort. Percent change from baseline in **(a)** partially exhausted $C8^+$T cells ($KLRG1^+TIGIT^+$), **(b)** CD8+ T effector memory cells (T_{EM}, $CD3^+CD56^-CD8^+CD45R0^+CCR7^-PD1^+$) and **(c)** T central memory cells (T_{CM}, $CD3^+$ $CD56^-CD8^+CD45R0^+CCR7^+PD1^+$). Data are expressed as median (25th, 75th centile).
